# Clinical Studies on Ultrafractionated Chemoradiation: A Systematic Review

**DOI:** 10.3389/fonc.2021.748200

**Published:** 2021-11-16

**Authors:** Erica Scirocco, Francesco Cellini, Alice Zamagni, Gabriella Macchia, Francesco Deodato, Savino Cilla, Lidia Strigari, Milly Buwenge, Stefania Rizzo, Silvia Cammelli, Alessio Giuseppe Morganti

**Affiliations:** ^1^ Radiation Oncology, Istituto di Ricovero e Cura a Carattere Scientifico (IRCCS) Azienda Ospedaliero-Universitaria di Bologna, Bologna, Italy; ^2^ Department of Experimental, Diagnostic and Specialty Medicine—Alma Mater Studiorum Bologna University, Bologna, Italy; ^3^ Università Cattolica del Sacro Cuore, Dipartimento Universitario Diagnostica per immagini, Radioterapia Oncologica ed Ematologia, Roma, Italy; ^4^ Fondazione Policlinico Universitario “A. Gemelli” Istituto di Ricovero e Cura a Carattere Scientifico (IRCCS), Dipartimento di Diagnostica per Immagini, Radioterapia Oncologica ed Ematologia, Roma, Italy; ^5^ Radiotherapy Unit, Gemelli Molise Hospital, Catholic University of Sacred Heart, Campobasso, Italy; ^6^ Medical Physic Unit, Gemelli Molise Hospital, Catholic University of Sacred Heart, Campobasso, Italy; ^7^ Medical Physics Unit, Istituto di Ricovero e Cura a Carattere Scientifico (IRCCS) Azienda Ospedaliero-Universitaria di Bologna, Bologna, Italy; ^8^ Service of Radiology, Imaging Institute of Southern Switzerland, Ente Ospedaliero Cantonale (EOC), Lugano, Switzerland

**Keywords:** chemo-sensitization, low-dose radiotherapy, systematic review, clinical trials, combined modality treatment

## Abstract

**Aim:**

The efficacy of low-dose fractionated radiotherapy (LDFRT) and chemotherapy (CHT) combination has large preclinical but little clinical evidence. Therefore, the aim of this review was to collect and analyze the clinical results of LDRT plus concurrent CHT in patients with advanced cancers.

**Methods:**

A systematic literature search was conducted on PubMed using the PRISMA methodology. Only studies based on the combination of LDFRT (< 1 Gy/fraction) and CHT were included. Endpoints of the analysis were tumor response, toxicity, and overall survival, with particular focus on any differences between LDFRT-CHT and CHT alone.

**Results:**

Twelve studies (307 patients) fulfilled the selection criteria and were included in this review. Two studies were retrospective, one was a prospective pilot trial, six were phase II studies, two were phase I trials, and one was a phase I/II open label study. No randomized controlled trials were found. Seven out of eight studies comparing clinical response showed higher rates after LDFRT-CHT compared to CHT alone. Three out of four studies comparing survival reported improved results after combined treatment. Three studies compared toxicity of CHT and LDFRT plus CHT, and all of them reported similar adverse events rates. In most cases, toxicity was manageable with only three likely LDFRT-unrelated fatal events (1%), all recorded in the same series on LDFRT plus temozolomide in glioblastoma multiforme patients.

**Conclusion:**

None of the analyzed studies provided level I evidence on the clinical impact of LDFRT plus CHT. However, it should be noted that, apart from two small series of breast cancers, all studies reported improved therapeutic outcomes and similar tolerability compared to CHT alone.

**Systematic Review Registration:**

www.crd.york.ac.uk/prospero/, identifier CRD42020206639.

## Introduction

Conventionally fractionated curative radiotherapy (RT) is delivered in 1.8–2.0 Gy daily fractions. Conversely, low-dose fractionated RT (LDFRT) is defined as the use of very small dose per fraction (< 1.0 Gy). In some experimental models, LDFRT resulted more effective than predicted by the linear quadratic model in terms of improved cell kill (1, 2). In particular, *in vitro* experiments showed this phenomenon in several cell lines (3–5). Interestingly, the higher efficacy of LDFRT was confirmed in human cells by several laboratories using different assay techniques, conditions of cell growth, handling, and irradiation (1). On the contrary, a relative tumor cell radiation resistance was recorded when higher doses per fraction were used (6). The low-dose hyper-radiation sensitivity (HRS) phenomenon has been interpreted on the basis of a threshold effect in radiation-induced damage repair. In fact, DNA-repair mechanisms are triggered only above certain dose levels, while lower doses are ineffective in arresting irradiated cells in the G2 cell-cycle phase (7, 8).

The peculiar efficacy of LDFRT has been interpreted also on the basis of immunological mechanisms. For example, Klug and colleagues (9) reported that local LDFRT produces efficient recruitment of tumor-specific T cells in human pancreatic carcinomas with T-cell-mediated tumor rejection and prolonged survival in otherwise immune refractory spontaneous and xenotransplant mouse tumor models. The authors used one single fraction with doses ranging between 0.5 and 6.0 Gy. They observed that the number of intratumoral T lymphocytes was higher after irradiation with the lowest dose (0.5 Gy) (9). Based on this preclinical evidence, LDFRT was tested also in a clinical study (10).

Concurrent chemoradiation is a standard treatment option in several tumors since CHT is able to act as a radiosensitizer. Interestingly, when delivered as LDFRT, also RT may act as a chemosensitizer. This peculiar synergistic effect of LDFRT and CHT was demonstrated by several preclinical studies, in different cell lines, and using different drugs such as cisplatin, carboplatin, docetaxel, and paclitaxel (11–15). It is worth noting that LDFRT-induced toxicity is significantly lower compared to conventional fractionation or hypofractionation. This higher tolerability allows LDFRT to be associated with “full-dose” CHT, with a clear benefit in terms not only of local response but also of systemic tumor control (16).

Considering these aspects, interest in the combination of LDFRT with CHT in the clinical management of cancer patients grew. LDFRT was proposed as a new systemic agent labeled with an “r” (e.g., gemcitabine plus LDFRT: rG) (17). Although some preliminary studies suggested the effectiveness of this combination (16, 17), randomized trials, meta-analyses, and systematic reviews on this topic are lacking. Therefore, the aim of this review was to collect and analyze the results of LDFRT plus CHT, currently available in literature, in terms of tumor response, clinical outcomes, and treatment tolerability.

## Methods And Materials

Our systematic review protocol was registered (registration number: CRD42020206639) within the International Prospective Register of Systematic Reviews (PROSPERO, www.crd.york.ac.uk/prospero/) on 31 August 2020.

### Inclusion Criteria

Human studies of any design, without limitations in terms of the number of enrolled patients, and based on LDFRT plus CHT combination, were included. Studies based on LDFRT without concurrent CHT were excluded. No restriction about total delivered dose, biological effective dose (BED), and RT technique was imposed.

### Outcome Measures

We reported the main findings of the analyzed papers with particular focus on clinical tumor response, overall survival, and treatment-related toxicity. Moreover, any differences between LDFRT-CHT and CHT alone were recorded and reported.

### Bibliographic Search

We conducted a search based on PubMed from the earliest date to 20 May 2020. In our review, we considered only studies published in the English language. We used various combinations of the subsequent terms in PubMed such as low-dose, radiotherapy, ultra-fractionation, hyper-radiation-sensitivity, chemosensitization, concurrent, and chemotherapy. Finally, the following two search strategies were used in PubMed: i) low-dose[All Fields] AND (“radiotherapy”[Subheading] OR “radiotherapy”[All Fields] OR “radiotherapy”[MeSH Terms]) AND concurrent[All Fields] AND (“drug therapy”[Subheading] OR (“drug”[All Fields] AND “therapy”[All Fields]) OR “drug therapy”[All Fields] OR “chemotherapy”[All Fields] OR “drug therapy”[MeSH Terms] OR (“drug”[All Fields] AND “therapy”[All Fields]) OR “chemotherapy”[All Fields]); and ii) “hyper radiation sensitivity” OR ((“ultrafractionation” OR “ultrafractionated”) AND (“radiotherapy” OR “irradiation” OR “radiation”)) OR (“chemosensitization” AND (“radiotherapy” OR “irradiation” OR “radiation”)). We found 396 studies with the first strategy and 253 with the second one. We removed duplicates, and we made the first selection based on titles and abstracts. Moreover, a further search through the references of the selected studies was performed. After reading the full-text articles, six studies were excluded: three used the term “ultrafractionation” or “low-dose RT,” but the delivered dose/fraction was ≥ 1 Gy; two studies did not use LDFRT plus CHT combination, and one study reported duplicated patients. Finally, 12 articles fulfilled our criteria (16–27).

### Study Selection and Quality Assessment

We used the PRISMA guidelines as a guide to select the items to be included within the review (28, 29). Title, abstract, and keywords of the identified articles were independently analyzed by two researchers (ES, AZ), and disagreements were solved by the senior author (AM). Potentially eligible studies were retrieved, and full-text evaluation was performed based on the inclusion and exclusion criteria by two different authors (ES, AZ) with disagreements resolved by consensus-based discussion. Subsequently, the following data were collected independently by two authors (ES, MB) from each article, with disagreements resolved by the senior author (AM): authors’ name and year of publication, study design, accrual period, patients and setting, treatment (LDFRT and CHT), and main outcomes. Papers were evaluated based on the ROBINS-I Risk of Bias tool (30). Two reviewers (ES, AZ) assessed the quality of the included studies, and discrepancies were resolved on agreement.

## Results

### Search Results

**Figure 1:**
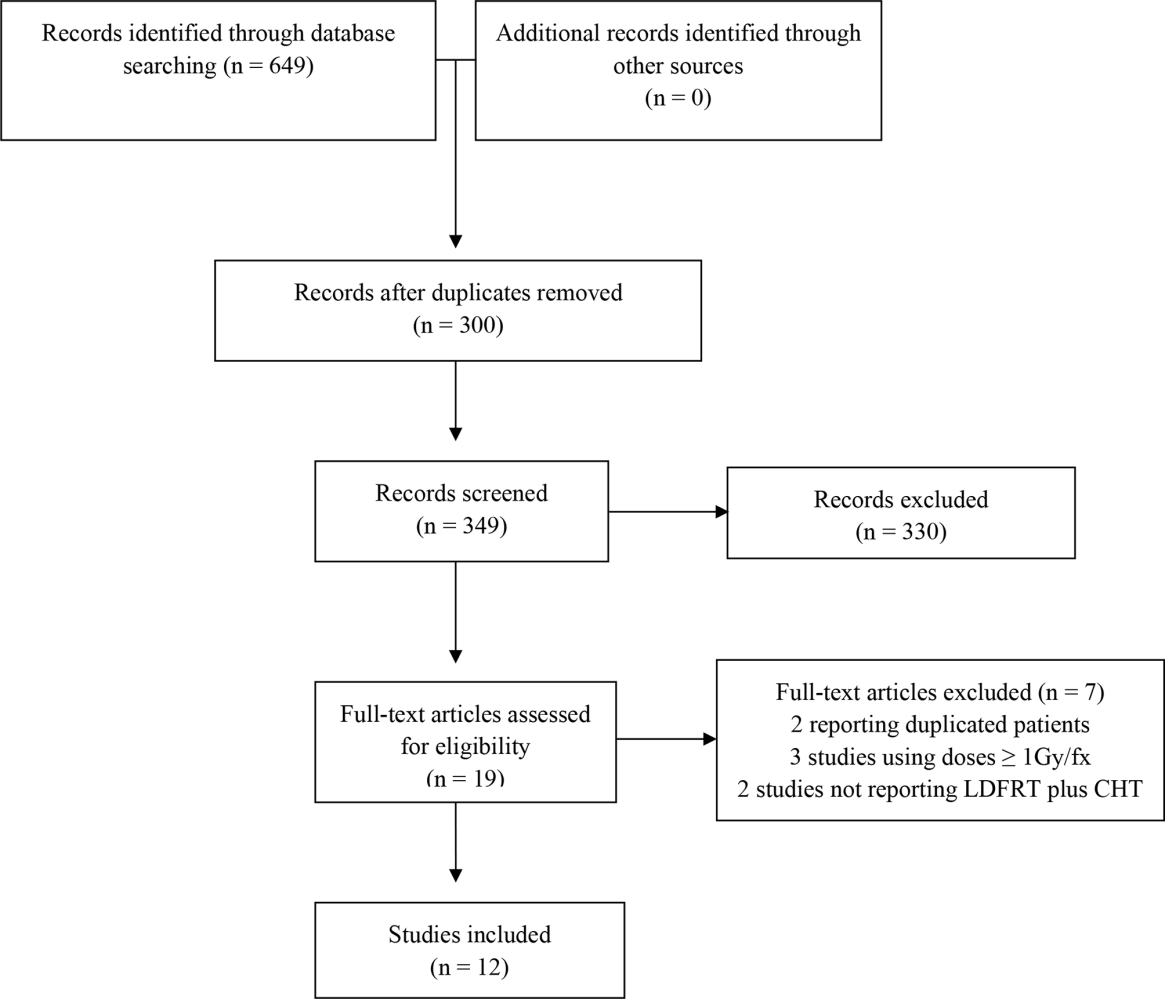
Process of paper selection.

Twelve articles (16–27) including 307 patients fulfilled the inclusion criteria for this review. Accrual period of all the studies ranged from 2000 to 2014. Details on the analyzed studies are reported in **Table 1**, while the flowchart of the literature search process is shown in **Figure 1**.

**Table 1:** Studies characteristics.

Study	Study design	No ofpatients	Median FUP	Setting	Treatment	
					Radiotherapy total dose (dose per fraction)	Chemotherapy
Arnold 2004 (16)	Phase II	40	18	Locally advanced SCCHN	3.2 Gy/4 fx (0.8 Gy, days: 1, 2, 22, 23)	Paclitaxel 225 mg/m^2^ i.v. (days 1, 22) + Carboplatin 10 mg/ml (within 30 min after Paclitaxel)
Regine 2007 (17)	Phase I/II	10	NR	Unresectable (5) or M1 pancreatic (liver) (4) or unresectable small bowel ca (1)	2 dose levels: 0.6 and 0.7 Gy/fx, BID, days: 1, 2, 8, 9. Four cycles planned	Gem 1,250 mg/m^2^ days: 1 and 8 at 10 mg/m^2^/min of a 3-week cycle
Valentini 2010 (26)	Retrospect.	22	6.5	Relapsed or metastatic ca of lung (12), H&N (7), breast (2); esophagus (1)	0.4 Gy BID repeated over 2 (lung, breast, and esophagus) or 4 (H&N) consecutive days, depending on the CHT schedule. Median total dose 8 Gy (range, 3.2–12.8 Gy).	Gem (1) or Cisplatin+Gem (1) or Pemetrexed (8) or Carboplatin (2) or Cisplatin+Fluorouracil (7) or Capecitabine (1) or Fluorouracil (1) or Docetaxel (1)
Mantini 2012 (21)	Phase II	19	6.5	Advanced NSCLC	1.6 Gy (0.4 Gy BID, days 1,2)	Concurrent Permetrexed 500 mg/m^2^ IV (cycles repeated fourfold every 21 days)
Nardone 2012 (24)	Phase I	10	NR	Breast cancer stage IIA/B-IIIA	0.4 Gy BID for 2 days every 21 days for 8–6 cycles	2 CHT schedules: 1) 4 cycles of nonpegylated liposomal doxorubicin sequentially followed by 4 cycles of docetaxel; 2) 6 cycles of nonpegylated liposomal doxorubicin + concurrent docetaxel
Nardone 2014 (25)	Phase II	21	31	Breast cancer stage IIA-IIIA	0.4 Gy BID, days: 1, 2, 6 of every cycle. First RT fraction delivered before CHT, the second fraction given at least 5–6 h later; cycle repeated every 21 days; total dose: 9.6 Gy (6 cycles)	6 cycles of liposomal anthracycline (50 mg/mq) and docetaxel (75 mg/mq) on day 1 of a 21-day cycle; cycle repeated every 21 days
Konski 2014 (20)	Phase I	27	8.4	Locally advanced or metastatic pancreatic cancer	3 RT dose level: 1) 28.8 Gy (0.4 Gy BID); 2) 28 Gy (0.5 Gy BID); 3) 28.8 Gy (0.6 Gy BID) days 1,2,8,9	Gem IV days 1, 8 + Erlotinib once PO (21 day cycles)
Balducci 2014 (18)	Prospective	32	22.5	Recurrent/ progressive GBM	Two schedules: 1) 0.3 Gy BID, days: 1, 2, 8, 9, 15, 16, every 42 days (2 cycles: total dose of 7.2 Gy); 2) 0.4 Gy BID over 5 consecutive days, every 28 days (2 cycles: total dose of 8 Gy)	Two schedules: 1) Cisplatin (30 mg/m^2^ on days 1, 8, 15) + Fotemustine (40 mg/m^2^ on days 2,9,16) if recurrent or progressive disease during adjuvant TMZ, on days 1, 2, 8, 9, 15, and 16, every 42 days; 2) TMZ rechallenge (150/200 mg/m^2^) if recurrent or progressive disease more than 4 months after adjuvant TMZ, over 5 consecutive days, every 28 days
Beauchesne 2015 (19)	Phase II	40	48	Newly diagnosed inoperable GBM	67.5 Gy/90 fx (0.75 Gy each 3 daily doses, at least a 4-h interfraction interval; 5 days a week)	Concurrent TMZ (dose of 75 mg/m^2^ for 7 days a week). At the end of a 4-week break, CHT was resumed for up to 6 cycles of adjuvant TMZ treatment, every 28 days according to the standard 5-day regimen.
Das 2015 (27)	Phase II	24	30	Locally advanced SCC of the cervix (stage IIB–IIIB)	3.2 Gy/4fx (0.8 Gy BID)	Paclitaxel (175 mg/m^2^) + Carboplatin (AUC X 5) 3 weekly for 2 cycles followed by radical chemoradiation
Morganti 2016 (23)	Phase II	18	30	Metastatic colorectal cancer	2.4 Gy (0.2 Gy BID, days: 1, 2 of every cycle)	12 FOLFIRI-B cycles (bevacizumab, irinotecan, bolus fluorouracil, and leucovorin with a 46-h infusion of fluorouracil, every 2 weeks)
Mattoli 2017 (22)	Retrospect.	44	NR	NSCLC (stage IIIA-IIIB)	100% patients: induction CHT + 0.4 Gy BID (days: 1,2 and 8,9 every cycle); 45% surgery; 59% neo-adjuvant CHT-RT (50.4Gy)	100% patients: 2 cycles of concurrent Platinum; 59% neo-adjuvant CHT+RT

FUP, follow-up; RT, radiotherapy; CHT, chemotherapy; SCCHN, squamous cell carcinoma of the head and neck; SCC, squamous cell carcinoma; RR, response rate; CR, complete response; PR, partial response; PFS, progression free survival; BID, bis in die; NRC, neoadjuvant radiochemotherapy; NAC, conventional neoadjuvant chemotherapy; PO, per oral; PMRR, pathological major response rate; TRG, tumor regression grade; GBM: glioblastoma multiforme, TMZ, temozolomide; Gem, gemcitabine.

### Study Design and Risk of Bias

Two studies were retrospective (22, 26), one was a prospective pilot trial (18), six were phase II studies (16, 19, 21, 23, 25, 27), two were phase I trials (20, 24), and one was a phase I/II open label study (17). No randomized controlled trials were found. All were considered to own moderate to serious risk of bias according to the ROBINS-I tool (30). **Appendix 1** shows the risk of bias rating per study based on the ROBINS-I tool.

### Analysis of the Selected Studies

#### Treated Tumors

The characteristics and stage of primary tumors in the analyzed papers are shown in **Table 1**.

#### Patients and Treatment

Patients’ median age ranged from 21 to 84 years (median 57.6) (16–18, 20, 22–26). Median follow-up ranged from 6.5 to 48 months (median: 22.5 months). The RT total dose ranged from 1.6 to 67.5 Gy. CHT was based on different schedules depending on tumor features. RT details and CHT schedules are shown in **Table 1**.

#### Evaluations

Response was reported in different ways in all the studies (16–27), while overall survival (OS) rates were reported in six studies (18, 19, 21–23, 27). Toxicity was reported in 11 studies (16–21, 23–27), mainly using the Common Toxicity Criteria for Adverse Events scale (31).

#### Treatment Results

Toxicity results are shown in **Table 2**. In most studies, the treatment was reasonably tolerated, despite obvious differences due to the different used CHT regimens (16–21, 23–27). In the phase II trial conducted by Beauchesne et al. (19) on LDFRT plus temozolomide in glioblastoma multiforme (GBM), three cases of fatal adverse events were reported: one due to hematological toxicity and two due to pulmonary infections. Moreover, Regine and colleagues (17), in their trial on gemcitabine plus LDFRT in pancreatic and small bowel cancers, reported one grade 3 infection out of six patients treated with 0.6 Gy/fraction and one grade 3 infection and one grade 3 diarrhea out of four patients treated with 0.7 Gy/fraction. **Table 3** reports details on tumor response and outcome. The results are very inhomogeneous as expected considering the different treated tumors and clinical settings.

**Table 2:** Toxicity.

Study	Main findings
Arnold et al., 2004 (16)	Grade 3,4 toxicities: neutropenia (50%), infection (8%), dermatologic reactions (8%), allergic reactions (3%), pulmonary reactions (3%), myalgia (3%). No grade 5 toxicity. Toxicity profile similar to CHT alone
Regine et al., 2007 (17)	1/6 experienced DLT at dose level 1 (0.6 Gy/fx): grade 3 infection; 2/4 experienced DLT at dose level 2 (0.7 Gy/fx): grade 3 nonhematologic infection and grade 3 diarrhea
Valentini et al., 2010 (26)	Grade 3–4 hematologic toxicities (9%); at a median follow-up of 6.5 months no local toxicity observed
Mantini et al., 2012 (21)	Neutropenia grade 4 (1 patient: 5.2%), already experienced during the prior CHT regimen (cisplatin and gemcitabine). Toxicity profile similar to CHT alone
Nardone et al., 2012 (24)	No grade 3, 4 toxicities. Toxicity profile similar to CHT alone
Nardone et al., 2014 (25)	No grade 2–4 hematological toxicities; no cardiac events
Konski et al., 2014 (20)	Very little > grade 3 toxicity; in cycle 4, one grade 5 bowel perforation in dose level 1 in one patient (3.7%) with a very large tumor with invasion of the duodenum; grade 3 ileus in the first cycle of therapy with dose level 1 in 1 patient (3.7%)
Balducci et al., 2014 (18)	Toxicities reversible without treatment-related death. Grade 2 fatigue (37.5%), grade 2 alopecia (50%), grade 1 skin reaction (9.3%), grade 1 headache (3.1%). Hematological toxicity (28.1%), with grade 1, 2 and 3, 4 in 18.7% and 9.4%, respectively. No late toxicity observed in retreated patients. LDFRT + CHT showed better toxicity profile when compared to the same group of patients treated with the different approaches available in this setting (re-resection, re-irradiation, different chemotherapy schedules)
Beauchesne et al., 2015 (19)	Fatal grade 4 hematological toxicity (2.5%), fatal pulmonary infection (5%)
Das et al., 2015 (27)	Grade 3, 4 hematological toxicity (24%)
Morganti et al., 2016 (23)	Grade 3, 4 toxicities 11.1%
Mattoli et al., 2017 (22)	Toxicity NR

RT, radiotherapy; LDFRT, low-dose fraction radiotherapy; CHT, chemotherapy; DLT, dose-limiting toxicity; NR, not reported.

**Table 3:** Response and outcome.

Study	Main findings
Arnold et al., 2004 (16)	ORR: 82% (assessed radiographically); RR: 90% at the primary site; RR: 69% at nodal site
Regine et al., 2007 (17)	ORR 30% (assessed radiographically); median OS 11 months (range: 4–37 months)
Valentini et al., 2010 (26)	ORR 45% (42% in previously treated patients); ORR of 57.1% and 41.6% in HN and lung cancer, respectively; with a median follow-up of 6.5 months no local toxicity observed
Mantini et al., 2012 (21)	ORR 42%; median OS 17 months. RR and median OS higher than CHT alone.
Nardone et al., 2012 (24)	50% clinical CR; TRG 1 (absence of residual cancer) 10%; TRG 2 (residual isolated cells scattered through fibrosis) 40%; PMRR 20% with LDFRT + sequential CHT and 40% with LDFRT + concurrent CHT
Nardone et al., 2014 (25)	PMRR: 33.3%; TRG1: 14.3%; TRG2: 19%
Konski et al., 2014 (20)	PR (30%), stable (55.5%), PD (3.7%); median OS 9.1 months
Balducci et al., 2014 (18)	CR 3.1%, PR 9.4%, stable disease 25% for at least 8 weeks after the end of treatment, 62.5% PD. Clinical benefit 37.5%. Median PFS and OS 5 and 8 months. Survival rate at 12 months 27.8%
Beauchesne et al., 2015 (19)	2y-OS 32.4%; 3-y OS 17.2%; median PFS 9.6 months; CR (10%); PR (17.5%). No improved OS (9.53 months) compared to unresectable GBM reported in literature
Das et al., 2015 (27)	OS and PFS at 2.5 years 84%. ORR (100% with 40% CR and 60% PR, based on MRI findings) and 3y-OS (80%)
Morganti et al., 2016 (23)	38.9% clinical or pathological CR; median OS 38 months; 2­y PFS: 63.9 and 31.2% and ORR: 83.3% and 33.3% in irradiated and not irradiated lesions, respectively
Mattoli et al., 2017 (22)	Response assessed by ^18^F-FDG PET-CT; at early PET-CT, 47.6% responders. At final PET-CT, 83% responders, 17.4% nonresponders (all nonresponders at early PET-CT). Early responders had higher PFS and OS than early nonresponders. Locoregional recurrence < 30%; 2-y OS rate was 59%; median OS 51 months

RT, radiotherapy; CHT, chemotherapy; LDFRT, low-dose fraction radiotherapy; ORR, response rate; CR, complete response; PR, partial response; PD, progression disease; PFS, progression-free survival; RECIST, response evaluation criteria in solid tumors; BID, bis in die (twice daily); GBM, glioblastoma multiforme; NRC, neoadjuvant radiochemotherapy; NAC, conventional neoadjuvant chemotherapy; PO, per oral; PMRR, pathological major response rate; TRG, tumor regression grade; HN, head and neck; ^18^F-FDG PET-CT, [18F]Fluoro-2-Deoxy-d-Glucose positron emission tomography/computed tomography.

#### Comparisons

Among all the studies included in our review, only Morganti and colleagues compared irradiated (LDFRT) and non-irradiated sites in patients with metastatic colorectal cancer treated with FOLFIRI-Bevacizumab (23). The authors reported 83.4% and 33.3% overall response rate (ORR) in irradiated and non-irradiated metastases, respectively (p: 0.02). Moreover, the 2-year progression rate was 63.9% and 31.2% in irradiated and non-irradiated sites, respectively (p: 0.08) (23). In other publications, the results of LDFRT-CHT were compared to those of CHT alone as reported in other studies (**Table 4**) (16–22, 24–27).

**Table 4:** Comparisons with chemotherapy alone.

Study	Main findings
Arnold et al., 2004 (16)	LDFRT + CHT well tolerated with higher RR delivering less CHT cycles compared to CHT alone. Toxicity profile similar to CHT alone
Regine et al., 2007 (17)	RR and survival rates higher than CHT alone
Valentini et al., 2010 (26)	ORR higher than CHT alone seen in different settings
Mantini et al., 2012 (21)	RR and median OS higher than CHT alone. Toxicity profile similar to CHT alone
Nardone et al., 2012 (24)	Toxicity profile similar to CHT alone
Nardone et al., 2014 (25)	PMRR was 33.3%, similar to CHT alone
Konski et al., 2014 (20)	Efficacy results compared to CHT alone (median OS of metastatic patients around 6 months in locally advanced disease with gemcitabine alone versus 9.1 months with LDFRT + CHT)
Balducci et al., 2014 (18)	LDFRT + CHT showed a very low toxicity profile when compared to the same group of patients treated with different approaches (36)
Beauchesne et al., 2015 (19)	Median OS of 16 months higher than OS rates reported in EORTC/NCIC trial (conventional RT + CHT versus conventional RT alone)
Das et al., 2015 (27)	ORR (100% with 40% CR and 60% PR, based on MRI findings) and 3y-OS (80%) with LDFRT + CHT followed by CHT + RT versus RR (70%) and 3y-OS (68%) with CHT + RT (the latter treatment scheme done with more CHT cycles). Lower toxicity grade with LDFRT+CHT followed by CHT + RT compared to treatment scheme using CHT+RT (the latter done with higher cycles of CHT)
Morganti et al., 2016 (23)	2­y PFS: 63.9 and 31.2%, ORR: 83.3% and 33.3% in irradiated and not irradiated lesions, respectively
Mattoli et al., 2017 (22)	Median OS higher than CHT alone

RT, radiotherapy; CHT, chemotherapy; SCCHN, advanced squamous cell carcinoma of the head and neck; HN, head and neck; ORR, response rate; CR, complete response; PR, partial response; PD, progression disease; PFS, progression free survival; RECIST, response evaluation criteria in solid tumors; BID, bis in die (twice daily); NRC, neoadjuvant radiochemotherapy; NAC, conventional neoadjuvant chemotherapy; PO, per oral; PMRR, pathological major response rate; TRG, tumor regression grade; DLT, dose-limiting toxicity.

## Discussion

To the best of our knowledge, this is the first review of clinical studies on combined LDFRT plus CHT. Five studies compared clinical response rates after LDFRT-CHT with literature data on CHT in similar patients, reporting higher ORR rates (16, 21, 23, 26, 27). Similarly, four studies compared OS after LDFRT-CHT and reported improved outcome compared to CHT alone (17, 19, 20, 22). Finally, four studies compared toxicity after LDFRT plus CHT versus CHT alone reporting similar adverse event rates (16, 21, 24, 25). Interestingly, clinical findings regarding LDFRT-CHT were published in 12 studies between 2004 and 2017, and no further studies were published thereafter. The lack of prospective studies, moreover with no control groups, could explain the disinterest in this combined modality therapy.

However, in most cases, the analyzed studies included only patients undergoing LDFRT plus CHT, without direct comparisons with patients undergoing CHT alone. In fact, differences were almost always tested against CHT results from other published studies.

Arnold et al. (16) reported higher ORR (90%), in advanced head and neck squamous cell carcinoma treated with LDFRT plus CHT, compared to literature data (55–75%) on similar patients treated with the same drug combination (carboplatin plus paclitaxel) (32–35). Regine et al. (17) reported prolonged OS after LDFRT plus gemcitabine, in locally advanced pancreatic adenocarcinoma, compared to literature data (36, 37) on gemcitabine alone (median OS: 11 months versus 4.8–5.6 months, respectively). Konski et al. (20) reported on locally advanced pancreatic cancer, with or without small burden metastatic disease, recording improved OS after LDFRT plus erlotinib and gemcitabine (9.1 months) compared to a study on erlotinib and gemcitabine alone (6.2 months) (38). Mattoli et al. (22) reported prolonged median OS in stage IIIA-IIIB non-small cell lung cancer treated with LDFRT plus concurrent induction CHT compared to another study (39) based on induction CHT alone in a similar patient population (median OS: 51 months versus 12.5 months, respectively). Beauchesne et al. published the results of their phase II trial (19) on inoperable GBM treated with LDFRT plus temozolomide reporting 16 months median OS. Surprisingly, this result is at least comparable with the outcome (median OS: 14.6 months) recorded in the EORTC/NCIC trial after standard RT plus temozolomide in patients with resected disease (40). Mantini et al. (21) reported 42% ORR and 17 months median OS in stage III-IV non-small cell lung cancer treated with LDFRT plus concurrent pemetrexed. These results were better compared to 9.1% ORR and 8.3 months median OS recorded in a similar patient population treated with pemetrexed alone (41). Valentini et al. (26) reported higher response rates in patients with lung (ORR: 41.6%) and head and neck cancer (ORR: 57%) treated with LDFRT-CHT compared to literature data on lung (ORR: 5–10%) (42, 43) and head and neck tumors (ORR: 10–35%) (44–47) treated with CHT alone (similar regimens). Das et al. (27) reported 100% ORR and 100% 2-year OS in locally advanced carcinoma of the uterine cervix treated with LDFRT plus induction CHT followed by radical chemoradiation. These figures were higher compared to the ones registered in a similar patient population treated with the same CHT induction regimen followed by standard chemoradiation (48). Only two studies did not show improved results after LDFRT plus CHT compared to CHT alone. In fact, Nardone et al. (24, 25) treated stage IIA/B-IIIA breast cancer patients with LDFRT plus CHT and reported similar response rates compared to CHT alone. However, it should be noted that the sample size of these studies was particularly small, with only 10 (24) and 21 patients (25) enrolled, respectively.

In terms of toxicity, Arnold et al. (16), Nardone et al. (24, 25), and Mantini et al. (21) reported similar toxicity profile in patients treated with LDFRT plus CHT compared to studies on CHT alone. Moreover, Balducci et al. (18) reported lower toxicity rates with LDFRT plus CHT compared to similar patient groups with recurrent GBM (49, 50) treated with several different approaches (second-line CHT, re-irradiation, re-resection). The worse complications recorded in the analyzed papers were reported in Beauchesne et al.’s (19) and Regine et al.’s studies (17). The first series included GBM patients treated with LDFRT plus temozolomide. Three cases of fatal adverse events were recorded: one after severe hematological toxicity and two due to pulmonary infections (19). It should be noted that these complications are not uncommon in patients treated with temozolomide alone. In particular, pneumonitis can occur when prophylactic treatment against pneumocystis carinii infections is not prescribed. In the second study, based on LDFRT plus gemcitabine in pancreatic and small bowel cancers, two grade 3 infections and one grade 3 diarrhea were reported (17). The irradiation of the entire upper abdomen could almost partially explain these adverse events.

A comparison within the same study between LDFRT-CHT and CHT was reported only by Morganti et al. As previously described, after CHT based on the FOLFIRI-bevacizumab regimen, the ORR rate was 83.4% in metastatic lesions undergoing LDFRT and 33.3% in non-irradiated lesions (p: 0.02) (23).

This review has several limitations including lack of randomized trials, heterogeneity of the study design with inclusion of two retrospective studies (22, 26), small sample size with a median number of 23 patients per study (range: 6-44) and four studies with less than 20 patients, and heterogeneity in terms of tumor and treatment characteristics. More specifically, the outcome results reported in two phase I (24) and phase I/II (17) trials, each enrolling only 10 patients, must be interpreted with caution due to the very small sample size. The usefulness of a literature review with these limitations could be debatable. However, due to lack of evidence from large prospective trials, we considered it useful to review the available data. Furthermore, it should be emphasized the uniformity between the analyzed series in terms of results, since all studies reported better outcomes after LDFRT-CHT compared to CHT alone, apart from two small studies on breast cancer (24, 25).

Based on the low level of evidence of the selected studies, the use of LDFRT-CHT in current clinical practice does not seem justified. However, especially in advanced cancers resistant to systemic therapies, enrollment of patients in prospective studies would be useful.

Further studies in this field could have the following design or aims: (i) randomized comparison between LDFRT-CHT versus CHT alone; (ii) definition of the optimal dose and fractionation in LDFRT-CHT; (iii) definition of the optimal CHT regimens in this setting; and (iv) evaluation of LDFRT plus immunotherapy combination, given some evidence on the immune-enhancement effect of LDFRT (51).

## Data Availability Statement

The original contributions presented in the study are included in the article/**Supplementary Material**. Further inquiries can be directed to the corresponding author.

## Author Contributions

Conception and design: ES, FC, AZ, and AM. Data extraction from included studies: ES, FC, GM, FD, and AGM. Analysis and interpretation of data: ES, FD, SCi, LS, MB, SR, SCa, and AM. Manuscript writing: all authors. All authors contributed to the article and approved the submitted version.

## Conflict of Interest

The authors declare that the research was conducted in the absence of any commercial or financial relationships that could be construed as a potential conflict of interest.

## Publisher’s Note

All claims expressed in this article are solely those of the authors and do not necessarily represent those of their affiliated organizations, or those of the publisher, the editors and the reviewers. Any product that may be evaluated in this article, or claim that may be made by its manufacturer, is not guaranteed or endorsed by the publisher.

## Appendix

**Appendix Table 1:** Overall risk of bias rating by study and corresponding reasons.

Component study	Overall “ROBINS-I Risk of Bias tool” judgment	Comments
Arnold et al. 2004 (16)	Serious	Bias in measurement of outcomes (one patient was removed from the study but included in the toxicity and response analysis; one refused additional chemotherapy after his first cycle but was analyzed in the treatment group)
Regine et al., 2007 (17)	Moderate	Bias due to confounding (heterogeneous setting of tumors)
Valentini et al., 2010 (26)	Moderate	Bias due to confounding (heterogeneous setting of tumors)
Mantini et al. 2012 (21)	Moderate	Bias due to confounding (heterogeneous setting of NSCLC)
Nardone et al. 2012 (24)	Moderate	Bias due to confounding (heterogeneous setting of breast cancer)
Nardone et al., 2014 (25)	Moderate	Bias due to confounding (heterogeneous setting of breast cancer)
Konski et al. 2014 (20)	Serious	Bias due to selection of participants into the study (select group of advanced pancreatic cancer patients with limited metastatic disease) Bias due to deviation from intended interventions (10/26 patients completed treatment; patients underwent chemotherapy schedule, which is currently reserved for those patients who cannot tolerate more intensive therapy)
Balducci et al. 2014 (18)	Moderate	Bias due to deviations from intended interventions (patients’ compliance was 78.1%)
Beauchesne et al. 2015 (19)	Moderate	Bias due to deviations from intended interventions (when tumor progression was found, patients were treated at investigator’s discretion)
Das et al. 2015 (27)	Moderate	Bias due to deviation from intended interventions (in 3 patients, delay in administered second cycle of low-dose fraction radiation therapy for personal reasons)
Morganti et al. 2016 (23)	Moderate	Bias in measurement of outcomes (3 patients underwent a subsequent resection of metastatic disease in the irradiated sites, rising the complete response rate up to 38.9% for irradiated lesions)
Mattoli et al. 2017 (22)	Moderate	Bias due to confounding (selection criteria not reported, heterogeneous setting of NSCLC and different strategy of treatment)
