# Supplementary material for: Clinical Studies on Ultrafractionated Chemoradiation: A Systematic Review
Source: Front Oncol. 2021 Nov 16;11:748200. doi: 10.3389/fonc.2021.748200 (PMC8635188; doi:10.3389/fonc.2021.748200)
Supplement: Supplementary file 1 [file DataSheet_1.pdf]

Appendix table 1: Overall risk of bias rating by study and corresponding reasons

Component study	Overall "ROBINS-I Risk of Bias tool" judgment	Comments
Arnold et al. 2004 (16)	serious	Bias in measurement of outcomes (one patient was removed from the study but included in the toxicity and response analysis; one refused additional chemotherapy after his first cycle, but was analyzed in the treatment group)
Regine et al. 2007 (17)	moderate	Bias due to confounding (heterogeneous setting of tumors)
Valentini et al. 2010 (26)	moderate	Bias due to confounding (heterogeneous setting of tumors)
Mantini et al. 2012 (21)	moderate	Bias due to confounding (heterogeneous setting of NSCLC)
Nardone et al. 2012 (24)	moderate	Bias due to confounding (heterogeneous setting of breast cancer)
Nardone et al. 2014 (25)	moderate	Bias due to confounding (heterogeneous setting of breast cancer)
Konski et al. 2014 (20)	serious	Bias due to selection of participants into the study (select group of advanced pancreatic cancer patients with limited metastatic disease)<br>Bias due to deviation from intended interventions (10/26 patients completed treatment; patients underwent chemotherapy schedule which is currently reserved for those patients who cannot tolerate more intensive therapy)
Balducci et al. 2014 (18)	moderate	Bias due to deviations from intended interventions (patients' compliance was 78.1%)
Beauchesne et al. 2015 (19)	moderate	Bias due to deviations from intended interventions (when tumor progression was found, patients were treated at investigator's discretion)
Das et al.	moderate	Bias due to deviation from intended interventions (in 3

2015 (27)		patients, delay in administered second-cycle of low-dose fraction radiation therapy for personal reasons)
Morganti et al. 2016 (23)	moderate	Bias in measurement of outcomes (3 patients underwent a subsequent resection of metastatic disease in the irradiated sites, rising the complete response rate up to 38.9% for irradiated lesions)
Mattoli et al. 2017 (22)	moderate	Bias due to confounding (selection criteria not reported, heterogeneous setting of NSCLC and different strategy of treatment)
